# Changes in the quaternary structure and function of MjHSP16.5 attributable to deletion of the IXI motif and introduction of the substitution, R107G, in the *α*-crystallin domain

**DOI:** 10.1098/rstb.2012.0327

**Published:** 2013-05-05

**Authors:** Roy A. Quinlan, Yan Zhang, Andrew Lansbury, Ian Williamson, Ehmke Pohl, Fei Sun

**Affiliations:** 1 Biophysical Sciences Institute, University of Durham, South Road, Durham DH1 LE, UK; 2 School of Biological and Biomedical Sciences, University of Durham, South Road, Durham DH1 LE, UK; 3 Department of Chemistry, University of Durham, South Road, Durham DH1 LE, UK; 4 National Laboratory of Biomacromolecules, Institute of Biophysics, Chinese Academy of Sciences, 15 Datun Road, Beijing 100101, People's Republic of China

**Keywords:** chaperone/small heat-shock proteins, desminopathy/crystallinopathy, IXI motif, structure and function, CRYAB/αB-crystallin/R120G, cryo-electron microscopy

## Abstract

The archael small heat-shock protein (sHSP), MjHSP16.5, forms a 24-subunit oligomer with octahedral symmetry. Here, we demonstrate that the IXI motif present in the C-terminal domain is necessary for the oligomerization of MjHSP16.5. Removal increased the *in vitro* chaperone activity with citrate synthase as the client protein. Less predictable were the effects of the R107G substitution in MjHSP16.5 because of the differences in the oligomerization of metazoan and non-metazoan sHSPs. We present the crystal structure for MjHSP16.5 R107G and compare this with an improved (2.5 Å) crystal structure for wild-type (WT) MjHSP16.5. Although no significant structural differences were found in the crystal, using cryo-electron microscopy, we identified two 24mer species with octahedral symmetry for the WT MjHSP16.5 both at room temperature and at 60°C, all showing two major species with the same diameter of 12.4 nm. Similarly, at room temperature, there are also two kinds of 12.4 nm oligomers for R107G MjHSP16.5, but in the 60°C sample, a larger 24mer species with a diameter of 13.6 nm was observed with significant changes in the fourfold symmetry axis and dimer–dimer interface. This highly conserved arginine, therefore, contributes to the quaternary organization of non-metazoan sHSP oligomers. Potentially, the R107G substitution has functional consequences as R107G MjHSP16.5 was far superior to the WT protein in protecting β_L_-crystallin against heat-induced aggregation.

## 1. Introduction

The small heat-shock protein (sHSP) class of protein chaperone is an energy-independent class of chaperone that act as holdases when combating the potential of misfolded or damaged proteins to form aggregates. sHSPs are also assembly chaperones, associating with protein polymers, such as actin, microtubules and intermediate filaments to modulate both their oligomerization and their assemblies in cells. How such properties relate to the structure of sHSP oligomers is a key question.

HSP16.5 from *Methanocaldococcus jannaschii* (MjHSP16.5) was the first sHSP to be structurally characterized by crystallography [1]. The protein oligomerizes into a particle containing 24 identical subunits with octahedral symmetry. The crystal structure confirmed earlier protein structure predictions [2] regarding a central α-crystallin domain (ACD) forming a β-strand sandwich comprised eight β-strands (numbered 2–9) organized in an immunoglobulin-like fold that is flanked by the N- and C-terminal domains that dock onto hydrophobic sites on the β4/β8 edge of this β-strand sandwich at the fourfold symmetry axis of the oligomer. The β6-strand hydrogen bonds to the β2-strand of its partner subunit in the twofold axis, forming an inter-subunit composite β-sheet, a feature found in the crystal structures of other non-metazoan sHSP oligomers [1,3,4]. The β6-strand therefore contributes to the oligomerization of individual sHSPs [5–7]. The edge formed by the β2- and β7-strands is where one of the highly conserved arginine residues [8] locates: precisely, the one which when mutated in αB-crystallin (CRYAB, CTPP2, HSPB5; [9]) and αA-crystallin (CRYAA, CRYA1, HSPB4; [10]), causes cataract (R116C CRYAA and R120G CRYAB) and cardiomyopathy (R120G CRYAB).

Previous studies on the R120G mutation in CRYAB revealed an increase in oligomer size [11]. Moreover, the R120G CRYAB oligomer had lost both its temperature and pressure dependence for size, and was also compromised in its heat stability compared with wild-type (WT) CRYAB [11]. By comparing a series of substitutions at the R120 site, it was apparent that the dimer, via its intra- and inter-subunit interactions, was very important to oligomerization and particle size [11]. Furthermore, for MjHSP16.5, oligomerization and particle size can also be altered by inserting extra sequences in its N-terminal domain as seen for HSP16.5-P1N [12].

Oligomerization differs remarkably between metazoan and non-metazoan orthologues. Non-metazoan sHSPs classically form oligomers of a defined subunit number, but metazoan sHSP complexes are typically polydisperse [8,13]. In non-metazoan sHSPs, the β6-strand is integrated into its partner dimer subunit, but this does not occur in metazoan sHSPs because the loop separating the β6- and β7-strands is non-existent, and they are instead fused into one [14]. One highly conserved structural element within the sHSP family [2,8,13] that is critical to oligomerization and to sHSP polydispersity is the C-terminal domain and specifically the IXI motif, which because of its palindromic nature, allows two different binding modes into the groove of the β4/β8 edge of the ACD [15]. The position and structural function of the IXI motif was identified in the crystal structures of MjHSP16.5 [12,16] as well as in the crystal structure of wheat HSP16.9 [4]. The MjHSP16.5 IXI motif and C-terminal extension therefore links together the four and threefold units in the oligomer, and its deletion would be expected to be deleterious for oligomerization [16]. This notion is further supported by the findings that deletion of the IXI motif prevents oligomerization of both bacterial and plant sHSPs [17,18]. The mammalian sHSP, HSPB6, is naturally missing this motif and does not oligomerize, forming dimers instead [19]. For a mammalian sHSP that does possess an IXI motif, deletion of C-terminal sequences including the IXI motif in CRYAB (Q151X in CRYAB) not only prevents its self-oligomerization, but also destabilizes it, with the benefit of apparently increasing its binding to client proteins [20]. Mutating the IXI to G-X-G in either CRYAB or CRYAA did not prevent oligomerization, but did improve its function in client protein protection assays [21]. Indeed, solution nuclear magnetic resonance (NMR) studies have shown that the flexibility of the IXI motif, and the C-terminal extension, in general, is critical to its function and point mutations that alter this flexibility change the activity of the sHSP [22]. Therefore, it is clear that this motif is key to sHSP protein oligomerization, dynamics and function, and it is important to formally confirm that this key role for the IXI motif is preserved in MjHSP16.5.

In this study, we analyse the effect of deleting the IXI motif and substitute the highly conserved arginine (R107) equivalent to the disease-causing mutation in CRYAB (R120G) into MjHSP16.5. In addition, we present the crystal structure for R107G MjHSP16.5 and by cryo-electron microscopy document a temperature-dependent effect upon its quaternary structure and assess the functional consequences by *in vitro* chaperone assays.

## 2. Material and methods

### (a) Cloning, mutagenesis and bacterial expression of MjHSP16.5

*Methanocaldococcus jannaschii* (Mj) DNA was obtained from Dr Huber (Microbiology, Regensburg, Germany). The MjHSP16.5 cDNA was generated by polymerase chain reaction (PCR) amplification using the primers 16.5FOR1: CATATGTTCGGTCGTGA CCCATTTGATTCATTATTTTG and 16.5REV1: TTATTCAATGTTGATTCCTTTCTTAATTGAGG using Pfu DNA polymerase (Fermentas, UK). PCR products were gel purified and cloned into pGEMT-Easy (Promega, UK). The cDNA was then subcloned into pET23b, using the *Nde*I and *Eco*RI sites *prior to* bacterial expression and purification after isopropyl-1-thio-β-d-galactopyranoside (IPTG) induction. The R107G mutation was created by PCR. The oligonucleotide pairs HSP16.5F1 and HSP16.5Rg1: AAGCTTTATTGTTCCATATATTTCTTCCTCTTCTG, and then HSP16.5Fg1: AAGCTTCCTGCAACTGTTAAGG paired with HSP16.5REV1. This strategy introduced the G107 mutation along with a distal *Hind*III site. After cloning both fragments into pGEMT-Easy, *Nde*I–*Hind*III, *Hind*III–*Eco*RI fragments were then ligated together into pET23b cut with *Nde*I and *Eco*RI to generate a bacterial expression vector to produce R107G MjHSP16.5. Lastly, a C-terminal truncation construct was produced, HSP16.5-G143STOP, using the primer pair MjHSP16.5Fstop1 and MjHSP16.5Rstop2 TTATTTCTTAATTGAGGATTCTGCCTTTGG. PCR fragments were then cloned into pGEMT-Easy. After blue-white selection after all the cloning steps detailed earlier, plasmids from individual clones were purified and both DNA strands sequenced, to confirm amplicon/ligation fidelity, and compared with the relevant database entry (http://srs.ebi.ac.uk/srsbin/cgi-bin/wgetz?-e+[EMBL_features-id:L77117_606]).

The expression constructs of both WT and engineered (R107G, G143X) MjHSP16.5 were transformed into BL21(DE3) pLysS *Escherichia coli*. Recombinant protein expression was induced using 0.5 mM IPTG for 4 h, once the bacterial culture had reached an OD_600_ of 0.6. Harvested pellets were resuspended in 50 mM Tris–HCl pH 8, 1 mM EDTA, 300 mM NaCl, 1 mM DTT, 0.2 mM PMSF containing complete protease inhibitor cocktail (Roche) and lysed by several freeze/thaw cycles. A supernatant fraction containing the soluble MjHSP16.5 was prepared by centrifugation of the lysate at 15 000 rpm in a JA-20 rotor (Beckman) for 30 min at 4°C after incubation for 30 min with 10 U ml^−1^ of benzonase nuclease (Merck Bioscience) at 4°C followed by 0.005% (w/v) polyethyleneimine for 10 min on ice.

### (b) Purification of MjHSP16.5 proteins

*Methanocaldococcus jannaschii* is a hyperthermophile and lives in hyperthermal oceanic vents, which experience temperatures of approximately 50–95°C and NaCl concentrations of approximately 0.2–1.0 M [23]. These organisms must therefore possess unique adaptations to allow survival in such high temperatures and salinity levels [24]. With this as a background, the soluble protein fraction was incubated in a water bath at 80°C for 30 min in the presence of 1 M NaCl. Samples were then cooled on ice for 10 min and centrifuged at 48 000*g* for 30 min. By using this approach, almost all contaminating proteins are denatured and pelleted, and the resulting soluble fraction was 90–95% MjHSP16.5. The protein was then further purified by anion exchange chromatography and size exclusion chromatography (SEC). Purification by anion exchange chromatography used a Fractogel EMD-650S TMAE column. Peak fractions were pooled and then dialysed into 20 mM Tris–HCl pH 7.4, 100 mM NaCl in preparation for SEC.

The R107G and G143X MjHSP16.5 proteins were purified using the same purification methods as WT MjHSP16.5, by heating and centrifugation, anion exchange and SEC. Purity was checked by sodium dodecyl sulfate-polyacrylamide gel electrophoresis (SDS-PAGE) and mass spectrometry as described [20]. For G143X MjHSP16.5, it was difficult to achieve more than 90 per cent. Although G143X MjHSP16.5 was heat stable, crystallization was not attempted. All pure protein samples were analysed by electron spray ionization mass spectrometry, and the measured molecular weights were within 2 kDa of the calculated molecular weight (data not shown).

### (c) Size exclusion chromatography of small heat-shock proteins

To estimate the molecular size of the recombinant sHSP complexes, approximately 5 mg of purified proteins were subjected to SEC. Purified MjHSP16.5 proteins were analysed using a Superose 6 column (290 × 10 mm) at a flow rate of 0.2 ml per minute at room temperature using a Merck–Hitachi biochromatography system. The column was calibrated with the following molecular mass standards: thyroglobulin (669 kDa), apoferritin (440 kDa), β-amylase (200 kDa), bovine serum albumin (67 kDa) and carbonic anhydrase (29 kDa). The void volume of the column was determined by dextran blue (2000 kDa). The proteins were separated on the column in buffer containing either 20 mM Tris–HCl, pH 7.4 or 10 mM HEPES, pH 7.4 and 100 mM NaCl at room temperature, and the elution volume of each sample was used to estimate the molecular weight. The data were analysed using Chromeleon 6.30 software (Sunnyvale, CA, USA).

### (d) Chaperone assays

Citrate synthase from porcine heart (Sigma-Aldrich, UK) was dialysed for 16 h at 4°C against 50 mM Tris–HCl pH 8.0, 2 mM EDTA and diluted to 0.49 mg ml^−1^. The assay mixture contained citrate synthase and MjHSP16.5 at a substrate : chaperone (4 : 1) ratio in 220 μl at a temperature of 42°C. The effectiveness of MjHSP16.5 as a chaperone at 60°C was measured using β_L_-crystallin as the client protein [25] and α-crystallin as a positive control. Both α-crystallin and β_L_-crystallin from bovine lens (Sigma-Aldrich) were dissolved in 50 mM sodium phosphate (pH 7.4), 100 mM NaCl. A chaperone-to-client ratio (1 : 1) by weight was used. Time-dependent light scattering was measured at 360 nm every 15 s over 30 min, using a Beckman DU640 spectrophotometer for both chaperone assays.

### (e) Crystallization

Initial crystallization experiments were performed using an Innovadyne screenmaker and standard sitting drop trays with drop sizes of 100 and 200 nl protein solution, respectively, mixed with 100 nl reservoir solution. Small crystals were observed in a number of conditions containing 2-methyl-2,4-pentanediol (MPD) and low molecular weight polyethylene glycol. The largest, best diffracting crystals were obtained from follow-up trays with hanging drop geometry using a reservoir solution of 0.02 M sodium acetate pH 5.5, 0.02 M CalCl_2_ and 35 per cent MPD as precipitating agents. Crystals grew over a period of weeks to maximum dimensions of 1.0 × 0.5 × 0.5 mm.

### (f) Data collection, processing and structure determination

Crystals were typically tested using the in-house Bruker MicroStar rotating anode equipped with a platinum 135 CCD detector [26]. All final diffractions were collected at the Diamond beam line I02 [27] and processed with XDS [28]. The structures of the WT and the R107G variant were solved by molecular replacement using the original MjHSP16.5 structure [1] with AMoRe [29], and refined using REFMAC [30]. All interactive modelling was performed with Coot [31]. Further details of the crystal structure determinations are summarized in table 1. Coordinates and structure factors for the R107G MhHSO16.5 have been deposited at the protein data bank (PDB ID: 4I88).

**Table 1.** Crystallographic dataset. Numbers in brackets refer to the highest resolution shell.

	wild-type	R107G
beam line	I02	I02
temperature (K)	100	100
wavelength (Å)	0.979	0.979
unit call *a* = *b* (Å), *c* (Å)	171.58, 175.58, 102.22	173.60, 173.60,103,0
resolution range (Å)	50–2.5	50–2.85
no. of observations	214 602	159 911
no. of unique reflections	38 818	27 031
no. of reflections (obs, %)	38 261	26 318
completeness (%)^a^	98.6 (98.3)	97.4 (99.7)
*I*/**σ**(*I*)	26.0 (2.7)	15.1 (4.0)
*R*_sym_	0.46 (60.0)	0.072 (0.49)
r.m.s.d bond lengths (Å)	0.015	0.015
r.m.s.d bond angles ( ˚)	1.55	1.44
*R*	0.199	0.195
*R*_free_^a^	0.266	0.254

^a^*R*_free_ calculated with five per cent of all reflections [32].

### (g) Cryo-electron microscopy

The final concentrations of both R107G and WT MjHSP16.5 used for cryo-electron microscopy (cryoEM) study were 0.8 mg ml^−1^. The sample (3.5–4.5 µl) was applied to the GIG holey grids (LifeTrust, PR China) after the girds were treated with a glow-discharge machine (Master Plasmer). Then, the samples were blotted for 3 s with blot force three at 100 per cent humidity using the VitrobotMark IV (FEI, The Netherland). The temperature of the Vitrobot chamber was set to room temperature and 60°C, respectively, for both the WT and the R107G MjHSP16.5 proteins. The grids were fast frozen into liquid-nitrogen-cooled liquid ethane.

The frozen samples were imaged with a FEI 300 kV Titan Krios cryo-electron microscope equipped with a Gatan UltraScan4000 (model 895) 16-megapixel CCD. A nominal magnification of 96 000× corresponding to the pixel size 0.933 Å and a defocus range between −2 and −3 µm were used during data collection for the WT MjHSP16.5 both at room temperature and at 60°C and for R107G MjHSP16.5 at 60°C. For the mutant R107G at room temperature, a nominal magnification of 75 000× was used, which corresponds to the pixel size 1.196 Å. The electron dose per image was kept around 20 e^−^ Å^−2^. For the R107G MjHSP16.5 at room temperature, 126 raw CCD electron micrographs were collected manually. For R107G MjHSP16.5 at 60°C and the WT MjHSP16.5 at both room temperature and at 60°C, electron micrograph exposures were made with the automatic collection package Leginon [33]. In total, 1308 raw images were collected for the R107G MJHSP16.5 at 60°C, 1160 raw images for the WT at room temperature and 2119 raw images for the WT MjHSP16.5 at 60°C. The EM maps for both WT and R107G HSP16.5 have been deposited in EMDB (EMBD IDs: EMD–2288 for the first model of WT MjHSP16.5 at room temperature (figure 4*a*); EMD–2289 for the second model of WT MjHSP16.5 at room temperature (figure 4*b*); EMD–2290 for the first model of WT MjHSP16.5 at 60°C (figure 4*c*); EMD–2291 for the second model of WT MjHSP16.5 at 60°C (figure 4*d*); EMD–2292 for the first model of R107G MjHSP16.5 at room temperature (figure 4*e*); EMD–2293 for the second model of R107G MjHSP16.5 at room temperature (figure 4*f*); EMD–2294 for the first model of R107G MjHSP16.5 at 60°C (figure 4*g*); EMD–2295 for the second model of R107G MjHSP16.5 at 60°C (figure 4*h*)).

### (h) Image processing

The defocus and astigmatism of each micrograph were estimated with CTFFIND3 [34] and corrected using the ‘applyctf’ routine of EMAN1 [35]. Particles of R107G (14 702 and 12 870) at room temperature and at 60°C and particles of the WT MjHSP16.5 (21 001 and 38 220) at room temperature and at 60°C were picked out using a graphical processing unit-accelerated, template–based, particles selection program, Gautomatch, developed in Fei Sun's laboratory (to be published). The initial models for the earlier-mentioned four different samples were obtained by using ‘startoct’ in EMAN1 [35]. Two different levels of noises were added into each initial model to yield two additional models. Then for each sample, these three corresponding models were used for further competitive image alignment and model refinement by using ‘multirefine’ in EMAN1 [35]. During the three-dimensional reconstruction, the octahedron symmetry was imposed. The final reconstructed density map was further sharpened by application of an amplitude correction algorithm in the program BFactor [36]. CryoEM maps were segmented, displayed and fitted with atomic models, using UCSF Chimera [37]. The crystal structure of the WT MjHSP16.5 (PDB code 1SHS) was used to fit into the cryoEM maps using the UCSF Chimera package [37].

The diameters of the reconstructed HSP16.5 particles were measured using the rotation-averaged projection method. First, the reconstructed volumes were projected by the same azimuthal angle to get the corresponding projections. Second, the projections were rotation-averaged using ‘proc2d’ in EMAN1 [35]. Finally, the rotational-averaged projections were projected along the *y*-direction to obtain the respective one-dimensional profiles. The distance between the two minimal values of the one-dimensional profile was measured as the diameter of the reconstructed particle.

## 3. Results

### (a) Characterization of wild-type, R107G and G143X MjHSP16.5

**Figure 1.**
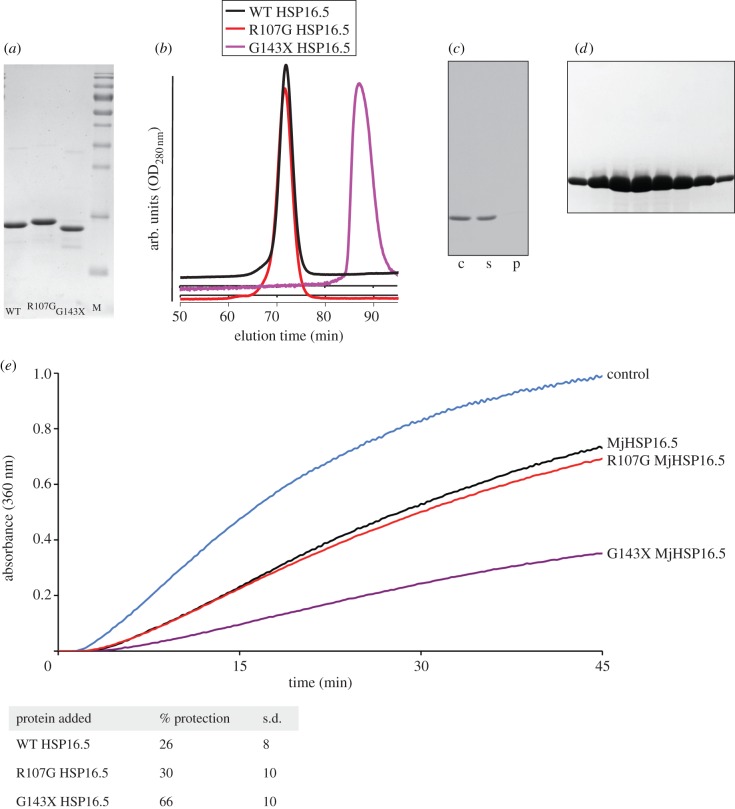
Characterization of the MjHSP16.5 proteins. (*a*) Purified samples of wild-type (WT) HSP16.5, R107G MjHSP16.5 and G143X HSP16.5 analysed by SDS–PAGE. Note the slower electrophoretic mobility of the R107G MjHSP16.5 sample. Page ruler markers (M) are included for reference. (*b*) Size exclusion chromatography (SEC) analysis using a superose 6 10/300 GL (GE Life Sciences, UK) column, which was calibrated using thyroglobulin (669 kDa), ferritin (440 kDa), aldolase (158 kDa), conalbumin (75 kDa) and ovalbumin (43 kDa) to produce a calibration curve (data not shown). Blue dextran (2000 kDa) was used to determine the void volume of the column. Both WT and R107G MjHSP16.5 had very similar elution profiles eluting with a calculated molecular weight of 402 kDa, whereas G143X HSP16.5 eluted in a volume equivalent to 34 kDa. (*c*) The post-SEC purified WT MjHSP16.5 was incubated at 80°C for 30 min (in the absence of NaCl) and then centrifuged at 48 000 *g*. More than 98% of the protein remains soluble, demonstrating thermostability of the WT HSP16.5 protein. Unheated (c), supernatant (s) and pellet (p). (*d*) SDS–PAGE analysis of the SEC peak for the purification of WT HSP16.5. (*e*) *In vitro* chaperone assay using the heat-induced aggregation of 0.2 g l^−1^ citrate synthase at 44°C. Results show that both WT and R107G MjHSP16.5 protect citrate synthase against heat-induced aggregation as measured by the change in optical density (360 nm) providing comparable levels of protections of 26% and 30%, respectively. G143X MJHSP16.5 (mauve line) showed significantly increased (approx. two times) protection of 66% compared with the WT protein and R107G MjHSP16.5 as summarized in the table. (Online version in colour.)

Wild-type MjHSP16.5 and the two engineered proteins R107G (the G107 substitution) and G143X MjHSP16.5 (the IXI motif deletion construct) were produced recombinantly in *E. coli* (figure 1*a*). The WT and R107G MjHSP16.5 were both purified using the modified protocol, which included a heating step (80°C for 30 min in the presence of 1M NaCl) to remove as many *E. coli* proteins as possible. Purification was completed by a final SEC step. Although it was previously found that at 60**°**C the protein tended to aggregate [38], this was not our experience (see below). R107G MjHSP16.5 was purified similarly, but the same degree of purification for G143X MjHSP16.5 was not possible and this prohibited detailed structural studies on this protein. SEC was used to analyse the oligomerization of the prepared MjHSP16.5 (figure 1*b*). WT and R107G MjHSP16.5 were very similar in their elution profile and consistent with a calculated oligomer of 24 subunits. Interestingly, the G143X MjHSP16.5 SEC characteristics were consistent with a dimer being the most prevalent oligomer in solution (figure 1*b*). WT MjHSP16.5 prepared in this way did not aggregate when heated to 80**°**C, the protein remaining in the supernatant fraction after sedimentation (figure 1*c*). SEC was also used to prepare the highly pure material for crystallization experiments (figure 1*d*).The *in vitro* chaperone activity of WT, G143X and R107G MjHSP16.5 proteins was determined using an assay based on the heat denaturation of citrate synthase (figure 1*e*). Results show that both WT and R107G MjHSP16.5 can protect against the heat-induced aggregation of citrate synthase, providing comparable protection (figure 1*e*). Interestingly, the G143X MjHSP16.5 protein provided significantly increased (approx. two times) protection (figure 1*e*).

### (b) Crystal structures of R107G MjHSP16.5 and comparison with the wild-type structure

**Figure 2.**
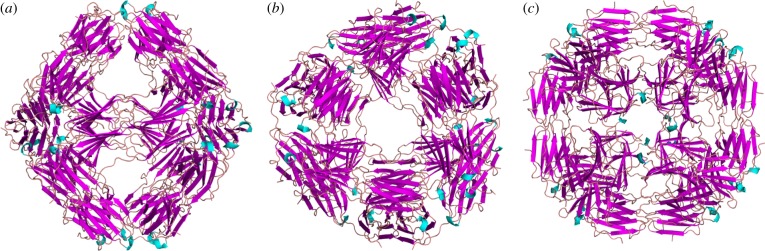
Ribbon diagram of the crystal structure of the MjHSP16.5 R107G. View shown along the (*a*) twofold, (*b*) threefold and (*c*) fourfold axis of the 24mer. The structure is isostructural to the WT MjHSP16.5.

**Figure 3.**
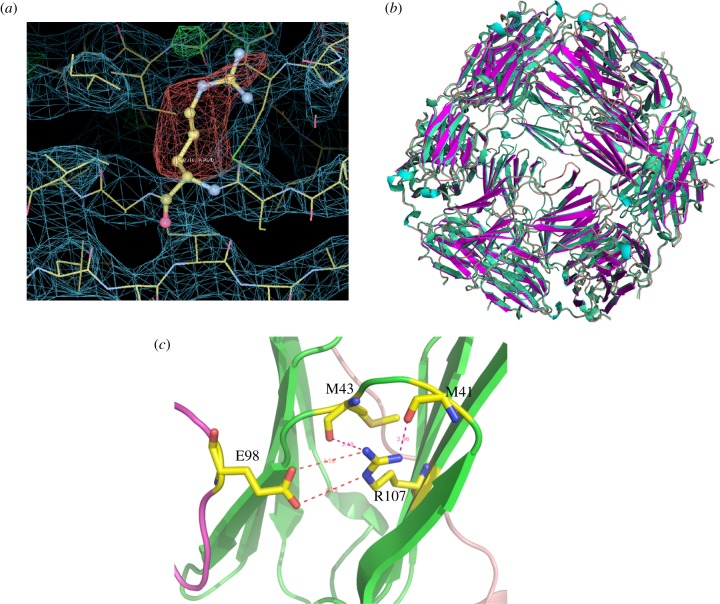
Crystallographic comparison of WT and R107G MjHSP16.5. (*a*) Initial 2*F*_o_ – *F*_c_ electron density calculated with molecular replacement phases using the WT MjHSP16.5 structures confirming the R107G substitution. Amino acid backbone (yellow) and the associated densities (blue) are shown with the R107G highlighted. The negative *F*_o_ – *F*_c_ density shown in red clearly indicated that R107 had been replaced. (*b*) Least-squares superposition of MjHSP16.5 WT determined at 2.5 Å (magenta) and R107G (cyan). Note that the crystal structures of the main chains are identical within the accuracy of the crystal structure determinations. (*c*) A close-up view of the mutation site with the R107 of the WT MjHSP16.5 and the loop residues shown in a ball-and-stick representation.

The R107G MjHSP16.5 crystallizes in the same rhombohedral space group with eight independent molecules per asymmetric unit as the WT protein [1]. The functional 24mer generated by crystallographic threefold axis is shown in ribbon representations in three different orientations (figure 2). Owing to the high diffraction data quality, the mutation is unambiguously confirmed even in the initial electron density map calculated with the molecular replacement solution using the WT structure. The negative electron density on all eight independent protein chains confirms the absence of side chain atoms (figure 3*a*). Clearly, the mutation does not cause any significant change in oligomeric state and overall structure (figure 3*b*).

Closer comparison between the higher resolution WT structure presented here and the R107G MjHSP16.5 shows that there are no significant changes in the main chain positions (figure 3*b*). The root-mean-square deviation of 6299 equivalent main chain atoms is only 0.45 Å—clearly within the accuracy of the crystal structure determination. Hence, even locally, there are no significant structural changes detectable. However, it is noteworthy that the R107 in WT MjHSP16.5 forms hydrogen bonds to the main chain carbonyl oxygen atoms of G41 and M43, and forms electrostatic interactions with the residue E98 of the partner chain (figure 3*c*). Although the R107G substitution does not affect the overall crystal structure of the protein in the crystal environment, the loss of these two H-bonds and the inter-molecular electrostatic interaction in the R107G MjHSP16.5 variant may very well change the dynamic behaviour in solution.

### (c) Cryo-electron microscopy reconstruction of wild-type and R107G MjHSP16.5 at room temperature and at 60°C

**Figure 4.**
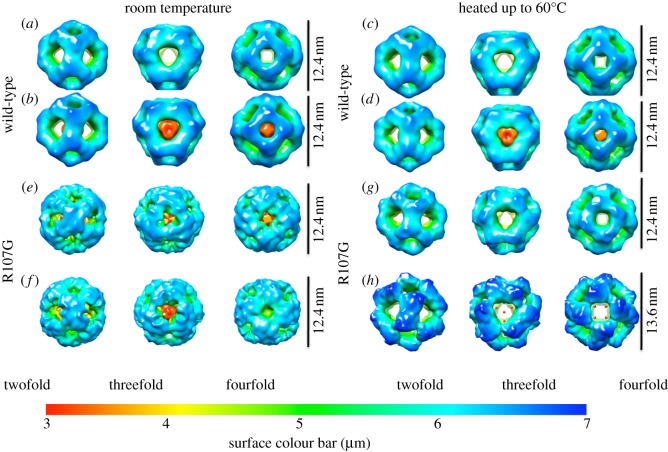
Cryo-electron microscopy maps of wild-type (WT) HSP16.5 and R107G mutant. (*a*,*b*) Two different structures for WT HSP16.5 at room temperature. (*c,d*) Two different structures for WT HSP16.5 heated to 60°C. (*e*,*f*) Two different structures for R107G mutant at room temperature. (*g*,*h*) Two different structures for R107G mutant heated to 60°C. All the structures are shown in three views along with the twofold, threefold and fourfold axis, respectively. The surface colours are varying from red to blue according to the distance to the centre from 3.0 to 7.0 nm. The diameter of each particle is labelled at the side.

The WT and R107G MjHSP16.5 incubated at room temperature and at 60°C were imaged and analysed by cryo-electron microscopic single particle reconstruction (see figure 4 and electronic supplementary material, figures S1 and S2). The final reconstructions of the WT MjHSP16.5 particle at room temperature converged to produce two major octahedral structures (figure 4). Although the diameters of the two structures are both 12.4 nm, the densities inside are different; one has an empty chamber (figure 4*a*), whereas the other is filled with very obvious central density (figure 4*b*). For the WT MjHSP16.5 at 60°C, there are also major octahedral structures obtained with the same diameter of 12.4 nm (figure 4*c,d*), similar to the room temperature particles. The final reconstructions of R107G MjHSP16.5 at room temperature were also converged to two structures with the same diameters 12.4 nm; one has an open structure around the fourfold axes (see figure 4*e* and electronic supplementary material, figure S1*a*) and the other has a closed structure at the same region (see figure 4*f* and electronic supplementary material, figure S1*b*). The chamber of both structures for R107G MjHSP16.5 at room temperature contains electron density.

**Figure 5.**
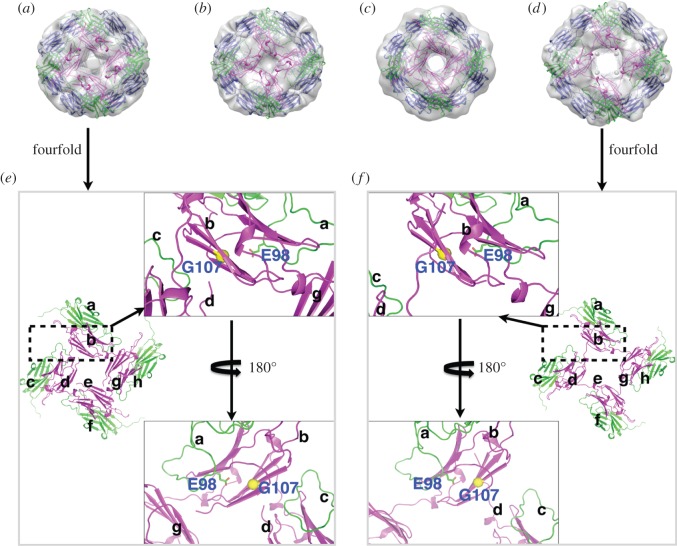
Fitting crystal structure of R107G MjHSP16.5 into cryo-electron microscopy maps. (*a*,*b*) Two different structures for R107G MjHSP16.5 at room temperature. (*c*,*d*) Two different structures for R107G MjHSP16.5 heated to 60°C. First, one dimer of the crystal structure of R107G MjHSP16.5 was fitted into the map along with the twofold axis. Then, all 24 copies of R107G MjHSP16.5 monomer were generated using the octahedral symmetry. (*e*,*f*) The close-up view of the dimer–dimer interfaces around the fourfold axis of the structures in (*a*) and (*d*), respectively.

The final reconstructions of R107G MjHSP16.5 at 60°C, however, revealed two significantly different structures with distinct diameters (see figure 4*g*,*h* and electronic supplementary material, figure S1*c*,*d*). The two different diameters of R107G MjHSP16.5 particles at 60°C were obvious after two-dimensional reference free classification (see the electronic supplementary material, figure S2). The smaller of the two particles is similar in size (diameter 12.4 nm) to the R107G and WT MjHSP16.5 particles at room temperature. Interestingly, the central chamber of this smaller (12.4 nm) particle for R107G MjHSP16.5 is empty in contrast to similar-sized particles at room temperature. The larger of the two, however, has a significantly increased diameter of 13.6 nm and was larger than any other structure derived in this study. These two reconstructions of the particles revealed different oligomer arrangements in the fourfold symmetry axis and the dimer–dimer interface for R107G MjHSP16.5 oligomers (see figure 5 and electronic supplementary material, figure S3). The dimer–dimer interface within the 12.4 nm diameter particle is equivalent to the arrangement in the crystal structures of WT (see the electronic supplementary material, figure S3) and R107G MjHSP16.5 (figure 5*a,e*). However, the distance between the dimers in the 13.6 nm particle is increased to reveal a large space at the dimer–dimer interface (figure 5*d,f*).

### (d) The R107G mutation alters the chaperone activity of MjHSP16.5 at 60°C

**Figure 6.**
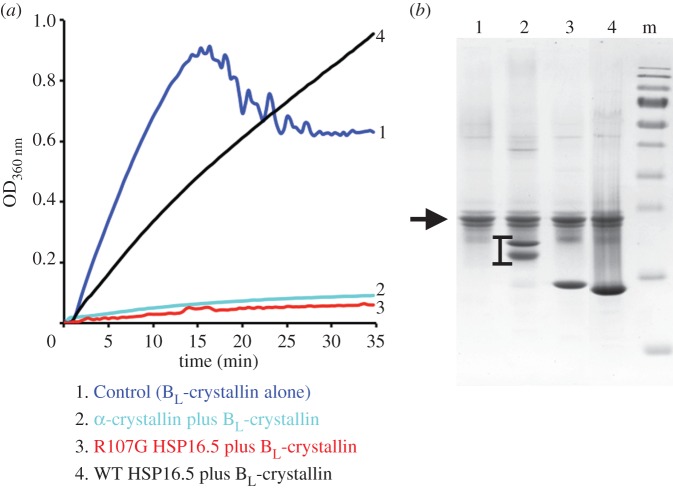
Comparison of the chaperone activity of WT and R107G MjHSP16.5 using a heat denaturation assay. (*a*) *In vitro* chaperone assay based upon the heat-induced aggregation of B_L_-crystallin at 60°C. Visible aggregates formed in the control (B_L_-crystallin alone) causing the trace to deteriorate in the final stages as these settled out in the sample. By contrast, the addition of equal α-crystallin prevented the optical density change and prevented the formation of large aggregates. A similar result was obtained with the addition of R107G HSP16.5, but this was not the case for WT HSP16.5. Lines each represent the average of three independent experiments. (*b*) SDS–PAGE analysis of the samples at the conclusion of the *in vitro* chaperone assay confirming the relative proportions of the chaperone additions to B_L_-crystallin (arrowhead). Note that α-crystallin (lane 2, bracketed) comprises two protein, αA-crystallin and αB-crystallin. R107G HSP16.5 is slightly slower in mobility (lane 3) compared with the WT protein (lane 4), both running just below the 15 kDa Pageruler marker band (lane m). (Online version in colour.)

The cryo-electron microscopy data suggest that the R107G substitution allows for some oligomer flexibility and therefore we considered whether this could influence the *in vitro* chaperone activity of MjHSP16.5. For this purpose, we used one of the earliest assays used to demonstrate the chaperone activity of sHSPs [25] because it could be conducted at 60°C and so be consistent with the cryo-electron microscopy analyses. As previously reported [25], α-crystallin very effectively prevented the heat-induced aggregation of β_L_-crystallin (figure 6*a*) when present in a 1 : 1 ratio (figure 6*b*). Similarly, R107G MjHSP16.5 was equally effective in suppressing the heat-induced aggregation of this artificial client protein. This is in stark contrast to the WT MjHSP16.5 (figure 6*a*), which appeared ineffective. Under the conditions of this assay and in the absence of an effective chaperone, β_L_-crystallin formed aggregates that over the course of the assay were large enough to settle under gravity, which is the reason for the variable signal towards the end of the assay. Clearly, the effect of the addition of MjHSP16.5 was quite distinct from that of either α-crystallin or R107G MjHSP16.5.

## 4. Discussion

### (a) The role of the N- and C-terminal domains on oligomerization of MjHSP16.5

Here, we have shown that removal of the C-terminal 5 residues from MjHSP16.5 is sufficient to significantly alter its oligomerization and also its chaperone activity towards citrate synthase (figure 1*e*). Our data are consistent with previous reports and with a key role for this domain in the oligomerization of MjHSP16.5 [16]. Preventing oligomerization by the removal of C-terminal residues mimic data obtained for CRYAB, where the myopathy-causing mutation Q151X in CRYAB prevented subunit oligomerization, but also increased its activity in *in vitro* chaperone assays [20]. Similar results are presented for MjHSP16.5.

Altering the N-terminal sequences in MjHSP16.5 can also increase the particle size with subunit number increased from 24 to 48 and also the client-protein-binding properties of the protein complex [12]. Swapping the N-terminal domain of CRYAB with that from CRYAA had a similar remarkable effect upon oligomerization and upon its chaperone function [39]. Similarly, deletion of the N-terminal domain prevents the oligomerization of mammalian sHSPs, for instance, CRYAB [15], and solid-state NMR studies of CRYAB demonstrate that this domain, which includes the β2-strand, interacts with the β3-strand of a neighbouring dimer [40]. Indeed, removing both the N- and C-terminal domains of CRYAB produces a stable ACD dimer that can be crystallized [14,15]. Therefore, it is clear that though there is significant sequence variation in the N- and C-terminal sequences between members of the sHSP family, they influence greatly sHSP subunit oligomerization and also their chaperone function [41].

### (b) Influence of the R107G mutation on the dimer interface and MjHSP16.5 oligomerization

Recent data suggest that the origin of metazoan sHSP particle size heterogeneity resides in the protein dimer and specifically the ACD [42]. The CRYAB ACD dimer can adopt different conformations as revealed by crystallography [14,15] and by solid-state NMR studies [40]. Mutations in the ACD that alter the dimer conformation would be expected to significantly change sHSP oligomerization, and one example of this is the disease-causing mutation R120G in CRYAB [42]. R120 in CRYAB is part of an extensive ionic interaction network that crosses the dimer interface and it influences the oligomerization properties of both CRYAB and CRYAA [43,44]. This arginine is a very highly conserved residue within the ACD domain of sHSPs [8].

As shown in this study, the introduction of glycine instead of arginine at position 107 had several interesting structural consequences for MjHSP16.5. In the first instance, the crystal structures revealed no significant difference in the electron density maps for the crystals of WT or R107G MjHSP16.5. This is likely due to the fact that in non-metazoans, the β6-strand exchanges between partner chains, which is absent in metazoan sHSPs [14,15,45], together with the crystallization conditions that constrained the oligomer packing. It was only by cryo-electron microscopy that structural differences around the dimer interface at the fourfold axis were observed and then only for MjHSP16.5 heated to 60°C. The WT MjHSP16.5 had two distinct classes represented in the samples prepared at room temperature and at 60°C, which is consistent with previous studies [38]. No changes in the dimer subunit arrangements were observable for these two different-sized particles. Indeed, the difference in these two particles was seen similarly for the R107G MjHSP16.5 at room temperature (figure 4). In both cases, the subunit compliment was unchanged (see figure 5 and electronic supplementary material, figure S3).

The dimer–dimer interface within the 12.4 nm diameter particle is equivalent to the arrangement in the crystal structures of WT (see the electronic supplementary material, figure S3) and R107G MjHSP16.5 (figure 5). The distance between the dimers in the 13.6 nm particle is increased to reveal a large space at the dimer–dimer interface. Each monomer has to adapt the conformation of its ACD loop to make appropriate interactions with the adjacent dimer. Such conformational change can be easily achieved as a result of the R107 substitution with glycine because this would abrogate the electrostatic interaction in the dimer between R107 in one monomer and E98 in an adjacent subunit to allow more flexibility in the ACD loop (figure 5), and this most likely is the reason why changes in the dimer interface can be accommodated without changing the subunit number in the assembled particle. This interpretation is consistent with the view that inter-subunit interactions are very important to oligomer size [11,42].

### (c) Influence of the R107G mutation on the chaperone function of MjHSP16.5

The R107G substitution in MjHSP16.5 also has functional consequences as evidenced by the increased chaperone activity towards β_L_-crystallin at 60°C (figure 6). The R120G substitution in CRYAB also changed its chaperone activity, either ablating completely or reducing it for the client proteins tested [43,44,46–48]. In CRYAB, there is an extensive network of ionic interactions that determine the AP interface between dimers [15], and these are disrupted by the R120G mutation in CRYAB causing the groove formed at the dimer interface to narrow [42]. This groove is also where small peptides bind in CRYAB [42]. If it is assumed that the equivalent interface in MjHSP16.5 can also potentially bind to client, then because this interface adopts a more open state at 60°C this would offer a potential explanation of its improved chaperone function.

Thermal-induced plasticity in chaperone activity combined with oligomerization has been observed for pea HSP18.1 [49]. HSP18.1 is a monodisperse dodecamer at ambient (22°C) temperatures, but heat shock (38°C) causes higher-order oligomeric states to form [49]. The addition of unfolding client proteins induces the formation of HSP18.1-client protein complexes that are remarkably polydisperse, varying in both the number of sHSP subunits and client proteins. WT and R107G MjHSP16.5 are constrained in their oligomerization dispersity. Nevertheless, the cryo-electron microscopy data indicate that R107G MjHSP16.5 forms at least two particles of different diameter, but of constant subunit number. Others have reported that WT MjHSP16.5 is also structurally diverse for the 24mer [38,50] and our data reveal two 12.4 nm particles differing in central density. The chaperone activity of MjHSP16.5 can also be increased when oligomer dispersity is driven by N-terminal sequence changes [12,16]. In the case of MjHSP16.5-P1 [16], there was no change in either the subunit number, the dimer interface or the symmetry, rather only a change in the orientation of the C-terminal sequences. This led to the suggestion that shell size for MjHSP16.5 depends on how the N-terminal sequences influence the C-terminal sequence orientation in MjHSP16.5-P1 [16]. The observed increase in client protein affinity and chaperone activity was reasoned to be due to the exposure of the N-terminal sequences [41,51]. We document here that preventing the oligomerization of MjHSP16.5 by deleting the C-terminal five residues increases *in vitro* chaperone activity, which parallels data obtained for CRYAB [20]. While our data do not distinguish between the two current models of sHSP-client protein recognition and binding [51,52], the increased chaperone activity towards β_L_-crystallin at 60°C as a result of the R107G substitution in MjHSP16.5 illustrates how such substitutions within the ACD can also contribute to structural and functional diversity of MjHSP16.5.

### Note added in proof

During the review of this manuscript, Shi *et al*. [53] reported cryo-electron microscope analysis for both WT and R107 MjHSP16.5. This study reported differences in the density distribution with the central cavity, which were interpreted to be due to changes in the relative position of the N-terminal domain as a result of the R107G substitution and indicating that R107G MjHSP16.5 was in an activated state. The data presented here are consistent with this interpretation, but only when the data for reconstructed particles at 60°C are considered. Moreover, our data demonstrate that the R107G substitution also facilitates the expansion of the ACD shell without the necessity of additional subunits.
